# Selective changes in vasopressin neurons and astrocytes in the suprachiasmatic nucleus of Prader–Willi syndrome subjects

**DOI:** 10.1111/jne.70015

**Published:** 2025-03-08

**Authors:** Felipe Correa‐da‐Silva, Jari B. Berkhout, Pim Schouten, Margje Sinnema, Constance T. R. M. Stumpel, Leopold M. G. Curfs, Charlotte Höybye, Ahmed Mahfouz, Onno C. Meijer, Alberto M. Pereira, Eric Fliers, Dick F. Swaab, Andries Kalsbeek, Chun‐Xia Yi

**Affiliations:** ^1^ Department of Endocrinology and Metabolism, Amsterdam University Medical Center, Location AMC University of Amsterdam Amsterdam The Netherlands; ^2^ Amsterdam Gastroenterology Endocrinology and Metabolism Amsterdam The Netherlands; ^3^ Department of Clinical Chemistry, Laboratory of Endocrinology Amsterdam University Medical Center, Location AMC Amsterdam The Netherlands; ^4^ Netherlands Institute for Neuroscience Amsterdam The Netherlands; ^5^ Dept. of Medicine Div. Endocrinology Leiden University Medical Centre Leiden The Netherlands; ^6^ Department of Clinical Genetics Maastricht University Medical Center Maastricht The Netherlands; ^7^ Governor Kremers Centre Maastricht University Medical Centre Maastricht The Netherlands; ^8^ Department of Endocrinology and Department of Molecular Medicine and Surgery Karolinska University Hospital and Karolinska Institute Stockholm Sweden; ^9^ Delft Bioinformatics Lab Technical University Delft Delft The Netherlands; ^10^ Dept. of Human Genetics Leiden University Medical Centre Leiden The Netherlands

**Keywords:** arginine‐vasopressin, Astroglial cells, biological rhythms, Prader‐Willi syndrome

## Abstract

The hypothalamic suprachiasmatic nucleus (SCN) hosts the central circadian pacemaker and regulates daily rhythms in physiology and behavior. The SCN is composed of peptidergic neuron populations expressing arginine vasopressin (AVP) and vasoactive intestinal polypeptide (VIP), as well as glial cells. Patients with Prader–Willi Syndrome (PWS) commonly experience circadian disturbances, which are particularly evident in their sleep/wake patterns. Using publicly available single‐cell RNA sequencing data, we assessed the cell‐type specificity of PWS‐causative genes in murine SCN, which revealed the differential presence of PWS‐related genes in glial and neural subpopulations. We then investigated neurons and glial cells in the SCN using immunohistochemistry in the postmortem hypothalami of PWS subjects and matched controls. We profiled neural populations characterized by AVP and VIP, astroglia characterized by glial fibrillary acid protein (GFAP), and microglia marked by ionized calcium‐binding adapter molecule 1 (Iba1) and NADPH oxidase 2 (NOX2). Our analysis revealed an increased total number, neuronal density, and relative staining intensity of AVP‐containing neurons in the PWS compared to controls while VIP‐containing cells were unaltered. In contrast, GFAP‐expressing astroglial cells were significantly lower in PWS subjects. Moreover, we did not detect any differences in microglia between PWS subjects and controls. Collectively, our findings show that PWS selectively affects AVP‐containing neurons and GFAP‐expressing astrocytes in the SCN. As each of these cell populations can affect the daily rhythmicity of the SCN biological clock machinery, the disruption of these cells may contribute to the circadian disturbances in patients with PWS.

## INTRODUCTION

Prader–Willi Syndrome (PWS) is a genetic imprinting disorder, mainly caused by a lack of expression of genes of the paternally acquired 15q11‐q13 chromosome region.^1^ PWS symptomatology is, for the major part, of hypothalamic etiology and includes, for instance, morbid obesity,^2^ hypogonadism,^3^ impaired growth, abnormal body composition,^4^ and behavioral aberrancies.^5^ PWS subjects also experience deteriorated circadian rhythm outputs, mostly evident through sleep disturbances,^6^, ^7^, ^8^, ^9^ concomitant to altered neuroendocrine^10^ and autonomic functions.^11^ To this date, the neuroanatomical basis, as well as pathophysiological mechanisms for these circadian disruptions reported in PWS remain unexplored.

The hypothalamic suprachiasmatic nucleus (SCN) constitutes the master circadian clock of the mammalian brain.^12^ SCN neurons control a multitude of physiological and behavioral daily rhythms, including neuroendocrine regulation,^13^ temperature fluctuations,^14^ and sleep–wake cycles.^15^ At the cellular level, SCN neurons generate rhythms through an autoregulatory feedback loop involving transcription factors that are termed clock genes. This ultimately translates into rhythms of gene expression with a period of approximately 24 h (i.e., circadian rhythms).^16^ The endogenously generated circadian rhythm of the SCN as a whole is synchronized to the exact 24 h rhythm of the environmental light–dark cycle by photic input from the retina via the retinohypothalamic tract.^17^, ^18^ Moreover, emerging evidence shows that SCN astroglia are involved in the proper synchronization of circadian rhythms generated within the SCN.^19^ Several lines of evidence suggest that circadian disruption is a symptom of numerous neurological and psychiatric disorders, including Alzheimer's disease (AD),^20^, ^21^, ^22^, ^23^ Parkinson's disease (PD)^24^, ^25^ Huntington's disease (HD),^26^, ^27^ and depression.^22^, ^28^ Numeric and functional alterations in the SCN circadian neural network have been reported in these pathologies, leading to defective generation of biological rhythms.^29^

To investigate whether the circadian disruptions of PWS subjects are associated with aberrant biological rhythm generation by their SCN, we resorted to publicly available hypothalamic single‐cell RNA‐sequencing (scRNA‐seq) datasets^30^ to investigate whether PWS‐causative genes are expressed in the SCN. Next, we profiled neural and glial markers in postmortem hypothalamic tissue of PWS subjects and controls.

## METHODS

### Single‐cell RNA‐seq resources

We used publicly available scRNA‐seq data from the murine hypothalamus atlas (HypoMap), and detailed information on experimental design and resources can be found in the respective original publication. Briefly, HypoMap is an integrated single‐cell atlas based on 17 studies of the mouse hypothalamus, generated using 10X Genomics Chromium and Drop‐seq. The dataset was acquired from CELLxGENE (HypoMap—a unified single cell gene expression atlas of the murine hypothalamus—CZ CELLxGENE Discover (cziscience.com)). First, the dataset was split into neuronal and non‐neuronal cells based on the “C7_named” annotation included in the HypoMap metadata. Neuronal cells were defined as “C7‐1: GLU,” “C7‐2: GABA,” and “C7‐6: ParsTuber”; the rest were classified as non‐neuronal. For the neuronal cells, an SCN subset was made based on the “Region_summarized” HypoMap annotation by selection for “Suprachiasmatic nucleus.”

**FIGURE 1:**
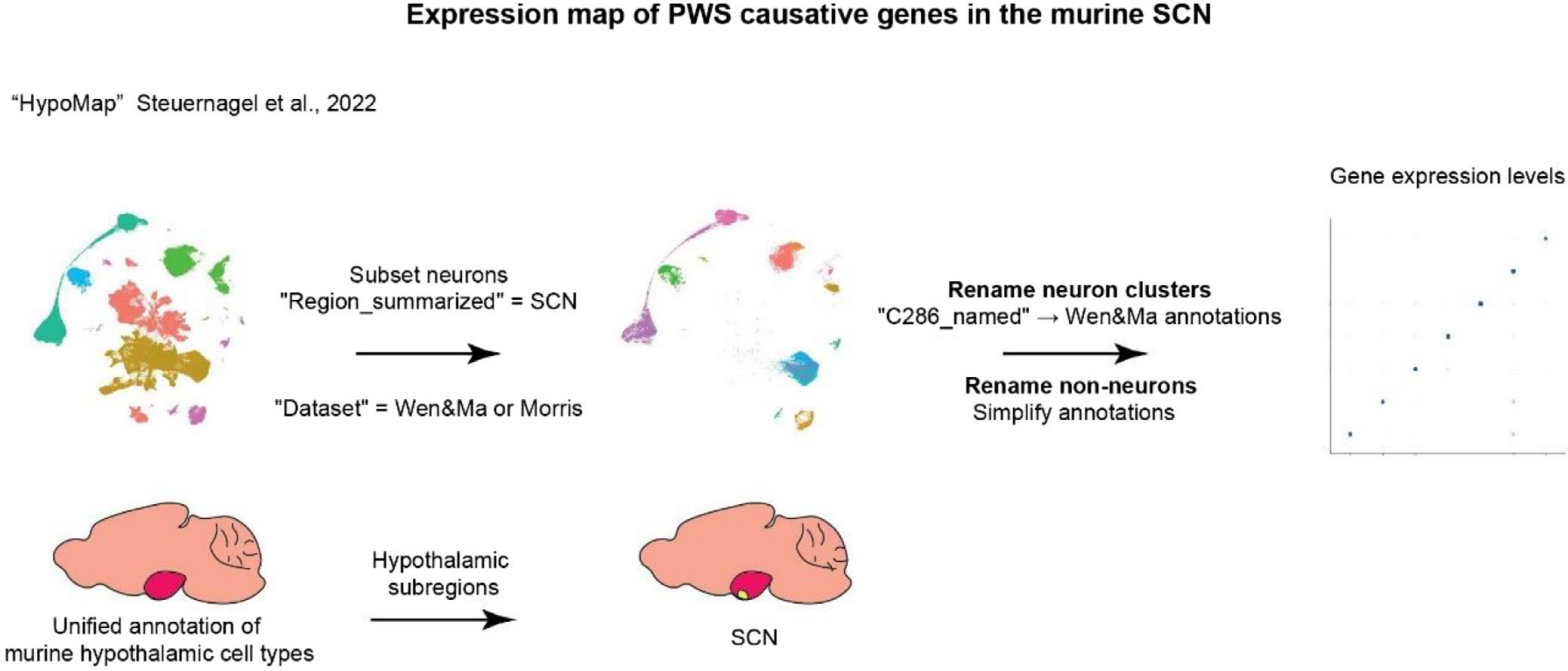
A schematic and simplified representation of the HypoMap interrogation pipeline, as detailed in the methodology section. In the illustration of the murine brain, the hypothalamus is highlighted in pink, while the SCN is shown in yellow.

For the non‐neuronal populations, SCN cells were selected based on the “Dataset” HypoMap annotation, by selecting cells annotated as “Wen10×,” “wenDropseq,” and “Morris10×” originally from the datasets of Wen and Ma (2020)^31^ and Morris et al. (2021).^32^ Subsequently, the resultant neuronal and non‐neuronal SCN subsets were merged into a unified SCN dataset. Using the HypoMap annotation “C66_named,” the non‐neuronal clusters were renamed to decrease the verbosity of annotations. For neuronal populations, the annotation level “C286_named” was used. These HypoMap‐defined clusters were renamed to match the annotations as previously defined by Wen and Ma.^31^ The matching of clusters to these annotations was based on the expression levels of the respective markers. The dataset was normalized with Seurat's NormalizeData function, using default parameters. Finally, the uniform manifold approximation and projection (UMAP) included with the HypoMap dataset was used for visualization. A schematic representation of the analysis pipeline is presented in Figure 1.

### Subject information

Postmortem hypothalamic tissues of 9 PWS subjects and 15 matched controls not diagnosed with PWS were obtained from the Netherlands Brain Bank (NBB). PWS subjects were matched to controls by gender, age, postmortem delay (PMD), month of death (MOD), and fixation time of the postmortem brain tissue (Table 1). Exclusion criteria for the controls included neurological and psychiatric disorders and the use of corticosteroids within 3 months prior to death. PWS chromosomal deletion was confirmed by Multiplex Ligation‐dependent Probe Amplification test.^33^, ^34^ Among PWS subjects, we identified two paternal deletion subtypes, namely seven individuals with type II deletions and two individuals with type I deletion that involves haploinsufficiency of an additional 4 protein‐expressing genes.^35^ All PWS subjects were profiled without dividing the genetic subtypes due to sample number limitation.

**TABLE 1:** Anthropometric and clinicopathological information.

	Sex	Age (years)	PMD (hours)	Fixation time (days)	MOD	BMI	Cause of death and clinical diagnosis
C 01	f	0,75	13	238	January	16	Sudden infant death syndrome
C 02	f	0,5	17	28	March	/	Cardiomyopathy
C 03	m	2	40	80	November	14	Severe post‐operative shock lung; hepatoblastoma
C 04	m	6	3,5	41	October	/	Intestinal necrosis and peritonitis
C 05	f	40	41	/	December	/	Pulmonary carcinoma
C 06	m	35	14	214	April	/	Respiratory insufficiency, sepsis due to pulmonary aspergillosis
C 07	f	65	20	35	February	27	Mesentrial ischemia
C 08	m	49	6,25	54	June	24	Euthanasia; Hodking's lymphoma
C 09	m	45	7	54	June	/	Brain hemorrhage
C 10	m	76	19	133	April	25	Septic syndrome; renal aneurysm
C 11	f	49	13,5	165	April	25	Respiratory insufficiency; cervix carcinoma
C 12	f	25	/	/	January	/	Epileptic insult
C 13	f	32	41	45	December	/	Pulmonary hypertension
C 14	f	77	2,55	39	March	33	Pulmonary metastases of vulva carcinoma
C 15	f	43	92	63	July	/	Unavailable
PWS 01	f	0,5	9,75	60	September	17	Positional asphyxia
PWS 02	f	30	4,83	/	November	52	Dyspnea, hypotension and acidosis
PWS 03	f	25	35	26	February	22	Cardiac arrest during surgery
PWS 04	f	33	5	31	June	71	Anuria and hypotension
PWS 05	m	32	48	59	December	/	Sudden death after fever and diarrhea
PWS 06	m	3	41	63	December	/	Unavailable
PWS 07	f	67	6,5	46	August	26	Pulmonary infection
PWS 08	m	49	/	/	July	/	Unavailable. Diabetes mellitus
PWS 09	m	50	43	/	July	/	Acute heart failure

Abbreviations: /, not available; BMI, Body mass index; C, controls; f, female; m, male; MOD, Month of death; PMD, Postmortem delay; PWS, Prader–Willi Syndrome; PWS, Prader‐Willi Syndrome.

### Immunohistochemistry and immunofluorescence

Immunohistochemistry and immunofluorescence were conducted according to previously published methods.^36^ In brief, during an autopsy, the dissected hypothalami were immersed in a fixative solution (10% phosphate‐buffered formalin) at room temperature. Hypothalamic tissue was ethanol‐dehydrated, toluene‐cleared, and paraffin‐embedded. The material was then coronally serially sectioned from rostral to caudal at 6 μm. Anatomical orientation of the SCN of the hypothalamus was determined by Nissl staining, referencing well‐defined anatomical landmarks, and confirmed by the presence of arginine‐vasopressin‐immunoreactivity (AVP‐ir).^37^ For each subject, every 50th section was evaluated for the neuropeptides AVP and vasoactive intestinal polypeptide (VIP). The section with the highest number of immunoreactive neuronal soma was identified as the “peak” for both AVP‐ir and VIP‐ir. Subsequent glia staining was performed on a neighboring section near the AVP‐ir peak. The following markers were studied in one section per subject: ionized calcium‐binding protein (Iba1) for microglia, NADPH oxidase 2 (NOX2) as a proxy for microglial activation, and glial fibrillary acidic protein (GFAP) for astrocytes. Antibody information is provided in Table 2.

**TABLE 2:** Antibody information.

Protein	Source	Host	Catalog number	Dilution	Antigen retrieval
AVP	NIN	Rb	Truss 86, C.P.230686	1/1000	pH 6.0
VIP	NIN	Rb	VIPER	1/1000	pH 6.0
Iba1	Synaptic Systems	Rb	234,003	1/600	pH 6.0
NOX2	Cell Sciences	Ms	CS‐MW1842	1/400	pH 6.0
GFAP	DAKO	Rb	Z0334	1/1000	pH 6.0
Secondary antibodies					
anti‐Rb (biotinylated)	Vector Laboratories	Hs	BA‐1000	1/400	
anti‐Ms (biotinylated)	Vector Laboratories	Gt	BA‐9200	1/400	
VECTASTAINABC Kit	Vector Laboratories		PK‐6100	1/800	
anti‐Rb 594	Invitrogen	Gt	A11037	1/400	

Abbreviations: Dk, donkey; Gt, goat; Hs, horse; Ms., mouse; NIN, Netherlands Institute for Neuroscience; Rb, rabbit; Stvd, Streptavidin.

Sections were deparaffinized in 100% xylene, rehydrated in grading ethanol (100%–50%), and rinsed in distilled water. Sections were washed in 0.05 M Tris‐buffered saline (TBS). Heat‐induced epitope retrieval using microwave treatment (10 min 700 W) was performed with Tris citrate buffer (pH 6.0). After cooling, sections were incubated with 3% hydrogen peroxide in SUMI incubation buffer (0.25% gelatin, 0.5% Triton X‐100 in 0.05 M TBS) for 10 minutes to eliminate endogenous peroxidase activity. Sections were then washed in TBS and incubated with primary antibodies for 1 h at room temperature and then overnight at 4°C. Next, sections were rinsed and incubated with biotinylated secondary antibodies. For immunohistochemistry, the product of the staining was visualized by incubation in 0.5 mg/mL 3,3′‐Diaminobenzidine (Sigma Chemical Co.) in TBS containing 0.01% H_2_O_2_. For immunofluorescence, the appropriate fluorescent secondary antibody was employed and DAPI counterstaining was performed (Table 2).

### Image acquisition and quantification strategy

Images of immunohistochemical staining were captured with an Axio Scanner (ZEISS) and then analyzed with QuPath software.^38^ Neuronal cell count was performed throughout the whole rostral–caudal axis of the SCN to determine the section with the highest number (peak) of AVP‐ and VIP‐ir neurons. Quantification for neuronal cell density and optical density measurements was performed in their respective peak sections, and glial parameters were analyzed in adjacent sections to the AVP‐ir peak. Quantification of AVP‐ir and VIP‐ir neurons was performed by manually outlining the area of coverage of the positive signal. Subsequently, the “pixel classification” and “intensity features” software tools were used to determine cell numbers and intensity of positive signals. Particles between 35 and 400 μm^2^ were considered positive soma, based on pilot studies (data not shown). The minimal soma size for neurons was determined based on the observation that with such a size a nucleus was still visible, similar to previously published material.^34^, ^36^, ^37^, ^39^ Total soma number was divided by the area of the outline, resulting in soma number/mm^2^. Quantification of microglial (Iba1‐ir and NOX2‐ir) and astrocytic (GFAP‐ir) markers was performed exclusively in the AVP‐ir area, since this is the most abundant neuronal population in the SCN. A similar quantification strategy to the one used for the neurons was employed for these markers. Due to the abundance of microglial/astrocytic processes, no minimum particle size was established. Estimation of total neuronal numbers was performed according to previously published protocols,^40^ and it was a result of multiplying the numerical cell density of each neural type with the corresponding volume of their area of coverage. Individual 1997–127 was excluded from the estimation of the total number of neurons due to the lack of material in the rostral part of the SCN. Individual 1986–004 was excluded from VIP‐ir neuronal density and relative intensity analysis as it did not show any VIP‐ir cells.

Images of immunofluorescent staining were acquired using a Leica SP8‐SMD confocal microscope. Co‐stained sections for GFAP and DAPI were acquired using a 10x lens. Laser intensity was determined in pilot studies and was unchanged during image acquisition. Similar to immunohistochemistry images, GFAP‐ir quantifications were performed in the AVP‐ir positive area of coverage, and no minimum or maximum particle size was determined. All quantifications were performed in a blinded manner. Digital image preparation was performed with Adobe Illustrator software (Adobe Systems, Inc., San Jose, CA, USA). No specific feature within an image was enhanced, obscured, introduced, moved, or removed.

### Statistical analysis

All data are expressed as mean ± SEM. D'Agostino and Pearson normality test was performed to determine data's normality. Comparisons were performed using an unpaired two‐tailed Student's *t* test or Mann–Whitney's test, for normal and non‐normal data distribution, respectively. Correlations were measured using linear regression. A *p*‐value less than 0.05 was considered significant. All statistical tests were performed using GraphPad Prism 9.

## RESULTS

### PWS causative genes are expressed in the mouse suprachiasmatic nucleus

**FIGURE 2:**
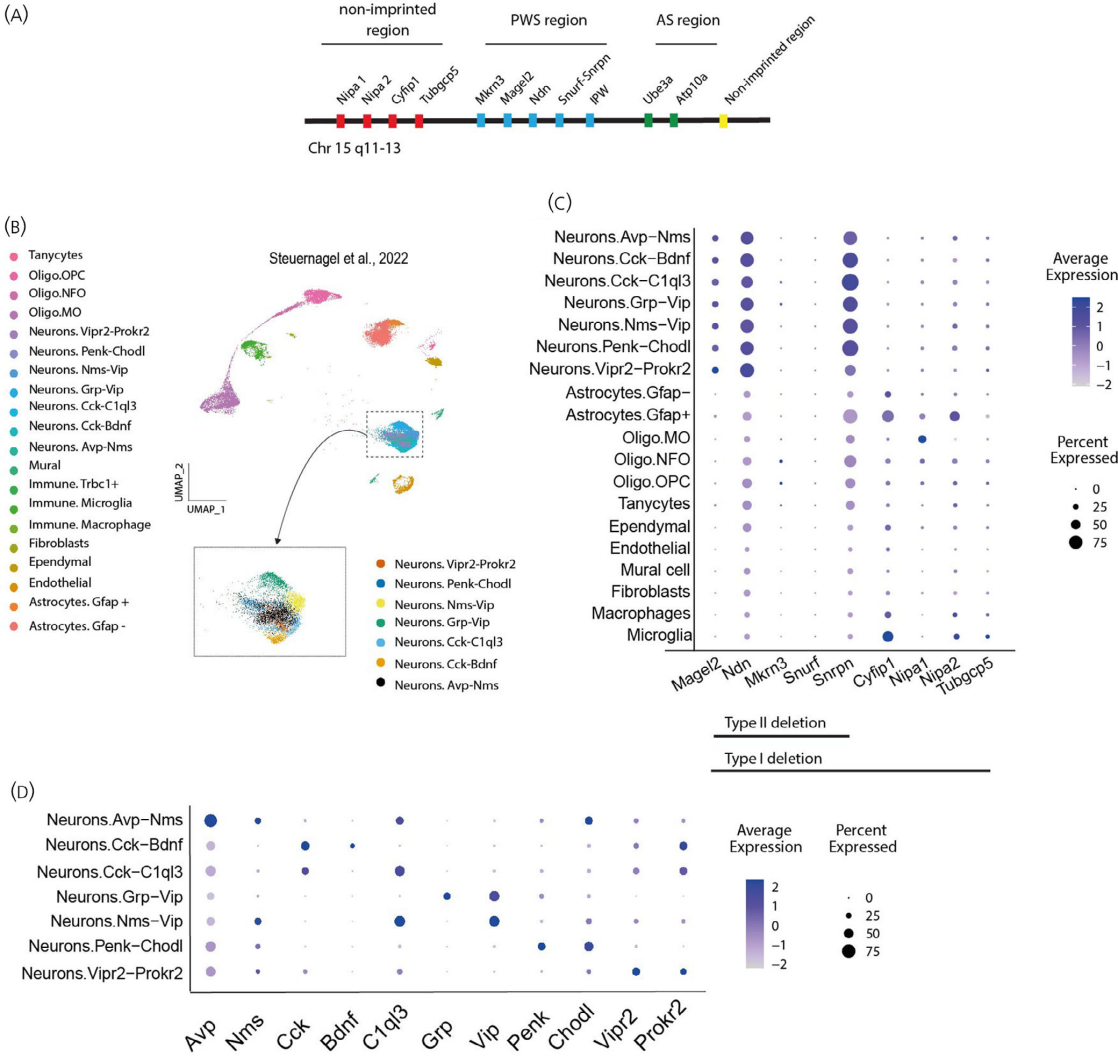
PWS causative genes are expressed in the SCN (A) Schematic representation of the chromosome map of the 15q11–13 region, illustrating the PWS causative genes. (B) UMAP of an SCN subset from the integrated single‐cell atlas HypoMap^30^ identifying 20 unique cell clusters. (C) Dot plot representing both the centered log‐normalized average expression (z‐score) and the percentage of positive cells for the PWS causative genes per cluster. (D) Dot plot representing both the centered log‐normalized average expression (z‐score) and the percentage of positive cells for the neuropeptides per cluster. PWS, Prader–Willi Syndrome; Chr, chromosome; Oligo, oligodendrocytes; OPC, oligodendrocyte precursor cell; NFO, Newly formed oligodendrocyte; MO, Myelinating oligodendrocyte; Gfap, glial fibrillary acid protein; Bdnf, Brain‐derived neurotrophic factor; Avp, Arginine‐vasopressin; Vip, vasoactive intestinal polypeptide; Vipr, receptor for vasoactive intestinal peptide; Penk, Proenkephalin; Prokr2, Prokineticin receptor 2; Chodl, Chondrolectin; Nms, Neuromedin S; Grp, Gastrin Releasing Peptide; Cck, Cholecystokinin; C1ql3, Complement C1q Like 3; Trbc1, T Cell Receptor Beta Constant 1; IPW, imprinted in Prader–Willi syndrome simplified representation of snoRNA‐associated region (including *Snord116*).

To understand which PWS causative genes (Figure 2A) are potentially implicated in the SCN neural and glial phenotype observed, we resorted to publicly available scRNA‐seq data from the murine SCN.^30^ The original HypoMap annotations were renamed and summarized for a total of 20 cell types (Figure 2B). Next, we evaluated the expression of protein‐coding genes present at the PWS critical genomic region (*Magel2*, *Ndn*, *Mkrn3*, *Snurf*, *Snrpn*). We also profiled PWS type I exclusive genes (*Nipa1*, *Nipa2*, *Cyfip1*, *and Tubgcp5*). Analysis of the scRNA‐seq data showed a specific expression pattern of PWS causative genes in different SCN cell types (Figure 2C). *Ndn* and *Snrpn* were expressed by most SCN subtypes but had higher expression in neuronal subtypes (Figure 2C). *Mkrm3* and *Snurf* were virtually not detected, with the exception of a small percentage of oligodendrocytes expressing *Mkrn3*. *Magel2* was almost exclusively expressed by neurons but was less abundant than *Ndn* and *Snrpn* (Figure 2C). An overview of the neuropeptidergic profile of SCN neuron subtypes analyzed is presented in Figure 2D. Interestingly, PWS Type I exclusive genes showed a glial signature, with three out of four genes having higher expression in GFAP‐ and Iba1‐expressing cells compared to the other genes analyzed. Taken together, these data indicate that PWS causative genes are expressed in different cellular domains in the SCN, with *Ndn* and *Snrpn* strongly represented in neural subtypes.

### Increased numbers of AVP‐containing neurons in the SCN of PWS subjects

**FIGURE 3:**
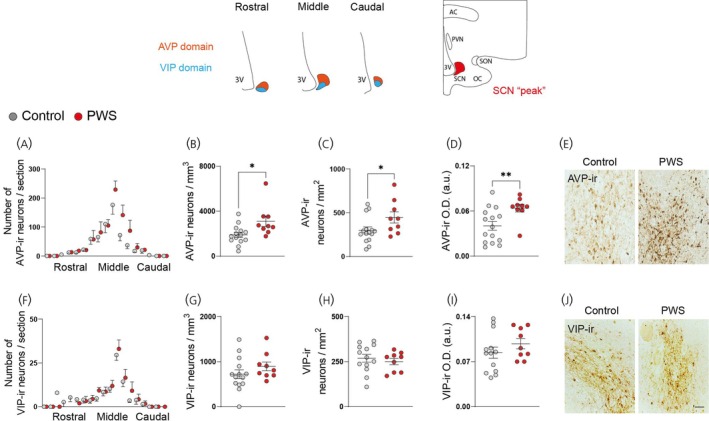
Increased numbers of AVP‐ir neurons in the SCN of PWS subjects. (A) The distribution of AVP‐ir neurons along the rostral to caudal axis of the SCN of controls and PWS individuals. (B) Total number of AVP‐ir neurons in the SCN of controls and PWS subjects. Quantitative analysis of (C) AVP‐ir soma number/mm^2^ (neuronal density) in the “peak” of the SCN and (D) AVP‐ir relative optical density measurements in the “peak” of the SCN. (E) Representative images of AVP‐ir neurons in the SCN of control and PWS subjects. (F) The distribution of VIP‐ir neurons along the rostral to caudal axis of the SCN of controls and PWS individuals. (G) Total number of VIP‐ir neurons in the SCN of controls and PWS subjects. Quantitative analysis of (H) VIP‐ir soma number/mm^2^ (neuronal density) in the “peak” of the SCN and (I) VIP‐ir relative optical density measurements in the peak of the SCN. (J) Representative images of VIP‐ir neurons in the SCN of control and PWS subjects. Please note the artwork detailing the SCN domains throughout the rostral–caudal axis. Additionally, illustration of a coronal hypothalamic section including the SCN “peak” and other anatomical landmarks. Controls (*n* = 15) and PWS (*n* = 9). Data are represented as mean ± SEM. *p < .05; **p < .001. Significance was calculated using Student's *t* test in B, C, G, H, I and Mann–Whitney test in D. Scale bar: 40 μm in E and J. 3v, third ventricle; AC, anterior commissure; PVN, paraventricular nucleus of the hypothalamus; SCN, suprachiasmatic nucleus; SON, supraoptic nucleus; OC, optic chiasm; AVP, arginine vasopressin; VIP, vasoactive intestinal polypeptide; PWS, Prader–Willi Syndrome.

As PWS causative genes are expressed in the murine SCN, we performed histological analysis of the major neuronal and glial populations in the SCN of PWS subjects and controls. First, we profiled the distribution of AVP‐ir and VIP‐ir along the rostral–caudal axis of the SCN. Overall, both PWS and control groups showed a similar range and distribution pattern of these neural cells (Figure 3A,F), indicating an intact SCN macrostructure. Of importance, total SCN volume was comparable between PWS and control subjects (AVP‐ir volume estimation 1.455 ± 0.999 vs. 1.128 ± 0.8493, *p* value = .6576; VIP‐ir volume estimation 0.555 ± 0.3252 vs. 0.5100 ± 0.4290, *p* value = .7673—values in mm^3^). However, the total numbers of AVP‐ir neurons were found to be significantly higher in PWS subjects in comparison to controls (Figure 3B). At the SCN peak level, we found that AVP‐ir neuron density was higher in comparison to control subjects (Figure 3C,E), accompanied by greater AVP‐ir staining intensity in PWS subjects (Figure 3D,E). Contrastingly, VIP‐ir total cell counts were comparable among both groups (Figure 3F,G). Likewise, cell density (Figure 3H,J) and VIP‐ir staining intensity (Figure 3I,J) in the peak of the SCN were unaltered in PWS subjects. These results indicate a neuropeptidergic imbalance in the SCN associated with PWS pathology, with a selective effect on AVP‐ir neurons.

### Decreased GFAP‐ir and unaltered microglial cells in the SCN of PWS subjects

**FIGURE 4:**
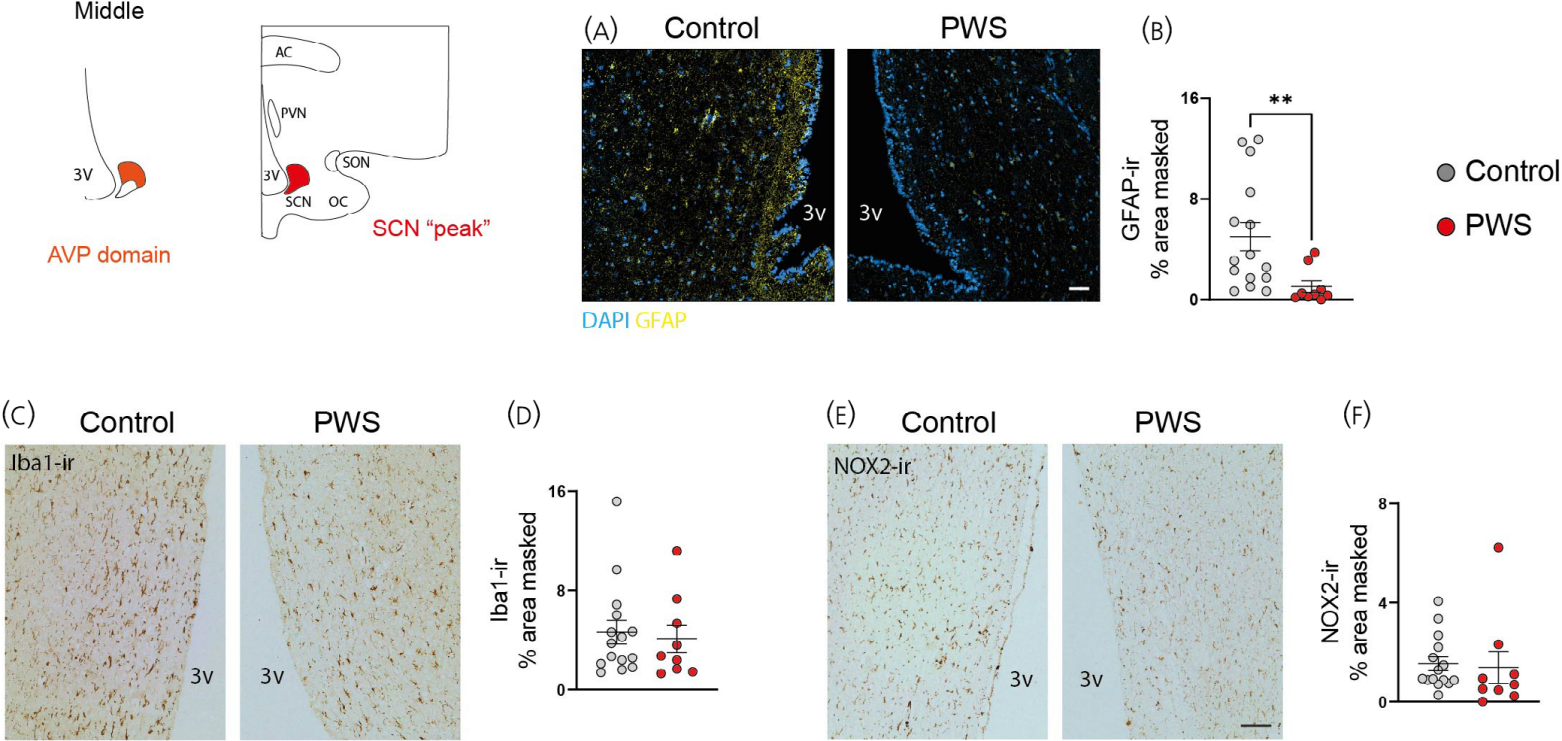
Reduced GFAP‐ir astrocytes and unaltered microglial cells in the SCN of PWS subjects. (A) Representative images of GFAP‐ir astrocytes in the SCN of control and PWS subjects. (B) Quantitative analysis of GFAP‐ir relative masked area. (C) Representative images of Iba1‐ir microglia in the SCN of control and PWS subjects. (D) Quantitative analysis of Iba1‐ir relative masked area (microglial cells). (E) Representative images of NOX2‐ir microglia in the SCN of control and PWS subjects. (F) Quantitative analysis of NOX2‐ir relative masked area. Data are represented as mean ± SEM. **p < .001. Significance was calculated using Student's t test. Scale bars, 50 μm in A, C, and E. Please note the artwork detailing the SCN “peak” domain, in which glial‐related quantifications were performed. This is accompanied by an illustration of a coronal hypothalamic section including the SCN “peak” and other anatomical landmarks. Controls (*n* = 15) and PWS (*n* = 9). 3v, third ventricle; AC, anterior commissure; PVN, paraventricular nucleus of the hypothalamus; SCN, suprachiasmatic nucleus; SON, supraoptic nucleus; OC, optic chiasm; AVP, arginine vasopressin; GFAP, glial fibrillary acid protein; Iba1, ionized binding protein 1; NOX2, NADPH oxidase 2.

Owing to the findings in AVP‐ir neurons, we investigated if non‐neuronal cells are also part of SCN neuropathology in PWS. To explore this, we profiled GFAP‐ir astroglia, which is also an essential coordinator of circadian timekeeping.^41^ We found reduced GFAP‐ir astrocytes in the PWS group compared to control subjects, evident through the lesser GFAP‐ir area of coverage (Figure 4A,B). Together, these results indicate a marked disruption in SCN neural and astroglial populations in PWS individuals.

To investigate whether AVP‐ir neuron changes were associated with increased local inflammatory events, we characterized SCN microglia cells. For this purpose, we performed a histological profile of Iba1 (microglial identifier, Figure 4C) and NOX2 (functional marker, Figure 3E). Both markers were unaltered in the PWS group (Figure 4D,F). These data indicate that disrupted SCN cytoarchitecture and circadian disruption in PWS are unrelated to microglia reactivity.

### Putative confounder analysis

Confounder analysis was performed on data for biological sex, age, fixation time, PMD, and BMI (Figures [Link], [Link]). Linear regression analysis revealed incidental significance for several parameters, including AVP‐ir soma number/mm^3^ and VIP‐ir soma number/mm^2^ versus BMI in controls: AVP‐ir soma number/mm and^2^ AVP‐ir O.D. versus age in controls; and Iba1‐ir % area masked versus BMI in PWS.

## DISCUSSION

A multitude of neuropathologies is associated with disturbances of circadian outputs, including PWS, in which sleep–wake disturbances are a clear clinical manifestation.^6^, ^7^, ^8^, ^9^ Yet, we lack insights into the fundamental neuroanatomical and pathophysiological mechanisms of circadian abnormalities in patients with PWS. Here, we report an imbalanced neuropeptidergic profile in the two major SCN neural populations and astrocytic alterations in the SCN of PWS subjects, the basis of providing some first insights on the cellular neuropathology and circadian disruption in the PWS.

The PWS genomic region (15q11–13 locus) contains several genes that encode either small nucleolar RNAs (snoRNAs) or proteins.^42^ Despite the circadian‐related phenotypes observed in PWS individuals, PWS causative genes have not been prominently studied in the context of SCN function. However, this does not preclude the possibility that these genes play a core role in SCN regulation. In fact, a putative role for *Magel2* in circadian timekeeping has been suggested, as *Magel2*‐null mice display abnormal onset of circadian behavioral activities.^43^ Later evidence demonstrated *Magel2* as a direct regulator of ubiquitination and stability of one of the core clock genes, cryptochrome circadian regulator (*Cry*).^44^ Besides *Magel2*, *Ndn* interacts with and stabilizes *Bmal1*, and *Ndn*‐null mice also display aberrant circadian behaviors.^45^ In addition, *Snord116* regulates expression and stability of another core clock gene, *Bmal1*, in a ubiquitin protein ligase E3A‐ (*Ub3ea‐*) dependent manner. Interestingly, recent evidence also demonstrated a critical role for Snord116 in the circadian regulation of the cortical methylome.^46^ Of importance, we were unable to quantify and visualize *Snord116*, coded by the IPW locus,^47^ probably due to the inherent difficulty associated with accurately sequencing and quantifying snoRNAs.^48^

To investigate the cellular domains of expression of PWS causative genes in more detail, we analyzed a comprehensive atlas of SCN transcriptional cell types at single‐cell resolution in the murine SCN from existing scRNA‐seq datasets.^30^ Our results demonstrate a strong neuronal signature of 3 genes, namely *Ndn*, *Snrpn*, and *Magel2*, reiterating the importance of *Magel2* and *Ndn* in circadian rhythm orchestration. *Magel2* is expressed almost exclusively by neurons, whereas *Ndn* and *Snrpn* are expressed in different glial cell types too, although to a lesser extent than in the neurons. Interestingly, despite the robust expression of *Snrpn* in the SCN, it was never investigated in the context of circadian regulation. It is noteworthy that a microdeletion of the SNURF‐SNRPN complex by itself is sufficient to cause a PWS‐like phenotype, such as obesity, dysmorphic features, and intellectual disabilities^49^; but whether these subjects display any circadian disruption is unknown.

Although PWS causative genes have not been prominently featured in SCN‐related studies, this does not preclude the possibility that these genes play a core role in the optimal functioning of the SCN.

There are two PWS subgenotypes, determined by the extent of the deletion.^34^, ^35^ PWS T1 subjects have a more extensive chromosomal deletion and present worsened clinical manifestations compared to PWS T2, evident in heightened compulsive behaviors and poorer cognitive function.^34^, ^35^, ^50^, ^51^ PWS T1‐associated genes showed a discrete expression in the SCN, mainly associated with *Gfap*‐positive astrocytes and microglial cells. Further studies are necessary to determine the impact of distinct PWS deletion types in circadian regulation, but our observations do not support strong deviations in different deletion‐associated subgenotypes. On the other hand, our results do support the idea that the expression of genes of the PWS critical genomic region is relevant for optimal neural functioning of the SCN.

The SCN is composed of a diverse neuronal network that is responsible for the synchronization of many physiological and behavioral circadian rhythms. AVP‐ and VIP‐containing neurons are the most abundant neuronal populations in the SCN of rodents^31^ and humans,^52^ with AVP‐expressing neurons being the pivotal pacesetter cells.^53^, ^54^ Numeric and functional changes in AVP‐containing neurons in the SCN translate into circadian misalignment, sleep disturbances,^55^ and, potentially, unbalanced hypothalamic autonomic output.^56^ Diminished numbers of AVP‐containing neurons in the SCN have been reported in a number of different diseases, including AD^57^ and type 2 diabetes mellitus (T2DM).^37^ Contrastingly, increased numbers were reported in subjects with mood disorders.^28^ Interestingly, in this mood disorder cohort, the authors also reported decreased levels of AVP mRNA, together indicating a defective neuropeptidergic transport and turnover in subjects with mood disorder. Imbalanced synthesis, maturation, and secretion of any neuropeptide directly impact their immunoreactivity pattern and therefore require careful interpretation. Here, we also report an increased number of AVP‐ir neurons in the SCN of PWS subjects. Of note, VIP‐containing cells were unaltered in the PWS group, indicating a selective effect on the AVP neuronal population, in spite of similar expression of PWS genes in (mouse) AVP and VIP neurons. Initial evidence points to the fact that PWS neurons have reduced neuropeptide production and neurosecretory capacity compared to controls,^58^ suggesting that the increased numbers we observed could be the result of abnormal AVP turnover and/or secretion in the SCN of PWS individuals.

Neural activity within the SCN generates target‐specific outputs that modulate a multitude of physiological processes, including energy homeostasis through neuroendocrine and autonomic modulation. SCN neurons innervate hypothalamic neurons that coordinate feeding behavior and energy expenditure,^59^ and SCN lesions lead to increased body weight and insulin resistance.^60^ Moreover, the SCN neural network is broadly recognized as a regulator of the autonomic nervous system,^61^, ^62^ and it is also able to interfere with visceral metabolism.^62^, ^63^, ^64^ Of importance, neuroendocrine^10^ and autonomic dysfunctions^11^ are reported in PWS subjects, but further research is necessary to understand the contribution of SCN circuitry to these events. Overweight and obesity prevalence in PWS exceeds 80%,^65^ which can be explained by a combination of endocrine abnormalities^66^ and defective neurocircuitry in the control of feeding behavior.^2^ In this context, considerable attention has been paid to neuronal populations that primarily control appetite, located in the infundibular and hypothalamic paraventricular nucleus.^34^, ^67^, ^68^, ^69^, ^70^ Examples of numeric loss and malfunctioning of these neurons have been reported in other metabolic disorders, such as T2DM,^36^, ^39^ sometimes concomitant with a loss of SCN‐residing neurons.^37^ Thus, it is reasonable to speculate that SCN defects in PWS contribute, at least partially, to the metabolic clinical manifestations observed in this pathology.

Compromised neuron–glia communication has been extensively reported in different neurological and brain‐related disorders,^71^, ^72^, ^73^ and it is considered a critical factor in the development and progression of hypothalamic dysfunction in metabolic disorders.^36^, ^74^ Curiously, our data indicate that neuropeptidergic alterations in the SCN of PWS subjects are not dependent on microglial function, similar to what is observed in T2DM.^37^ It is noteworthy that PWS was previously associated with a generalized decrease in GFAP‐expressing astrocytes in the dorsomedial hypothalamic nucleus,^69^ compatible with our findings in the SCN. Reduced SCN astrocyte numbers have been reported in pathologies associated with circadian imbalance, such as T2DM^37^ and AD.^20^, ^21^, ^22^, ^23^, ^57^ These findings in distinct pathological contexts suggest that astrocytes play a significant role in the SCN's homeostatic function.

Emerging literature demonstrates that astroglia actively shape and participate in the generation of biological rhythms. Therefore, PWS circadian desynchronization might be rooted in astrogliopathological events, at least partially. Astrocytic intrinsic clock coordinates astrogliotransmission, and it is a crucial component of rhythm generation in the SCN.^19^, ^41^, ^75^ Furthermore, genetic ablation of *Bmal1* in SCN astrocytes results in lengthened rhythms in the SCN and associated behavioral outputs.^76^ Interestingly, the astrocytic circadian timekeeping system is directly regulated by endocrine factors,^77^ suggesting that neuroendocrine imbalances might interfere with rhythm generation in an astroglia‐dependent manner. Additionally, astrocytic circadian defects have been previously linked to SCN integrity and sleep dyshomeostasis,^78^, ^79^ similar to the sleep fragmentation described in PWS pathophysiology. Compelling evidence shows that SCN‐residing astrocytes are intimately associated with AVP‐expressing neurons and directly affect their rhythm generation function.^41^, ^80^ Speculative interpretation of our results suggests that PWS pathophysiology is associated with intrinsic disruption in astroglia and AVP‐expressing neurons, along with astrocyte‐neuron communication disordering.

It is of essence to point out that we limited our analysis to the cardinal astrocytic marker GFAP. To which extent other astrocytic populations are affected by PWS pathophysiology remains to be explored. Curiously, our results show that PWS causative genes are expressed in a higher proportion of *Gfap*‐positive astroglia in contrast to *Gfap*‐negative in the murine SCN. Within the SCN, the loss of GFAP‐positive astrocytes indicates a major disruption in the pacemaker function in PWS, contributing to the observed neuronal impairments.

Taken together, our findings demonstrate a disruption in the SCN cellular composition in PWS. Our observations indicate the need for a deeper understanding of the association between circadian perturbations and PWS clinical outcomes. Whether the neuropeptidergic imbalance and reduced astroglia dictate circadian misalignments in PWS or emerge as a consequence of the disease pathology remains unknown. Observations in rodents and the effectiveness of circadian‐based therapies suggest the former,^81^ opening a potential therapeutic door for PWS symptomatology amelioration through pharmacological or behavioral approaches for circadian normalization.

### Limitations of this study

We acknowledge several limitations in our study. Extensive literature demonstrates that no animal model can fully recapitulate PWS pathophysiology.^82^ In our study, we took advantage of publicly available scRNA‐seq data to profile which SCN cell types expressed PWS causative genes. Since there is no available scRNA‐seq of the human SCN, we restricted our analysis to murine models. We cannot exclude potential distinct expression patterns of PWS‐related genes in both species. Second, it is important to recognize the limited size of our cohort, constrained by the rarity of PWS (prevalence of 1/10000 to 1/30000 in live births—www.pwsausa.org). Furthermore, PWS clinical complexity creates challenges for hypothesis testing and proper collection of well‐matched controls (i.e., as obese as PWS subjects). Our selection for controls in this study was rigorous, taking into consideration age, biological sex, MOD, medication prior to death, medical history, PMD, and tissue fixation time. Interestingly, our confounder analysis demonstrated that AVP‐ and VIP‐containing neurons are affected by age and BMI in control individuals. The limited number of PWS specimens and eligible matching controls (i.e., infants and young adults and/or as obese as PWS) precludes further investigation of the impact of this incidental significance in our cohort. It is noteworthy that matched correlations (e.g., AVP‐ir soma number/mm^3^ and AVP‐ir soma number/mm^2^; Iba1‐ir and NOX2‐ir area masked in PWS) did not show the same pattern of significance, probably indicating incidental significance.

Unfortunately, no detailed information on circadian disturbances was present in the medical records of PWS or control subjects. Furthermore, we lack detailed information on the time of death for the majority of subjects employed in this study, limiting our understanding of the impact of time of death on our selected markers. It is noteworthy that we previously demonstrated a lack of daily rhythmicity in AVP‐ir, VIP‐ir, and GFAP‐ir cells in the SCN of controls and metabolically diseased individuals (T2DM) in a previous study.^37^

Astroglia and microglia cells were profiled using pan glial markers (GFAP, Iba1, and NOX2). AVP‐ and VIP‐containing neurons were profiled throughout the whole SCN. Due to the relatively homogeneous distribution of glial cells in the brain, we conjectured that the results of astroglia and microglia in peak sections can reflect other domains of the SCN. This is reiterated by the fact that AVP‐ir and VIP‐ir volumes of coverage were unaltered in PWS subjects compared to controls. Therefore, we excluded the impact of the sagittal length of the hypothalamus or any other morphometric variation in our results. While glial functional and identity heterogeneity has been broadly recognized,^83^, ^84^ this diversity and its impact in the context of the SCN is virtually unexplored. Future research might elucidate the potential of astrocytic and microglial subpopulations in the generation of biological rhythms.

## AUTHOR CONTRIBUTIONS

**Felipe Correa‐da‐Silva:** Conceptualization; investigation; formal analysis; writing – review and editing; writing – original draft; visualization; validation; methodology. **Jari B. Berkhout:** Investigation; methodology; formal analysis. **Pim Schouten:** Investigation; formal analysis. **Margje Sinnema:** Writing – review and editing; resources. **Constance T. R. M. Stumpel:** Resources; writing – review and editing. **Leopold M. G. Curfs:** Writing – review and editing; resources. **Charlotte Höybye:** Writing – review and editing; resources. **Ahmed Mahfouz:** Supervision; writing – review and editing; methodology. **Onno C. Meijer:** Writing – review and editing; supervision; methodology. **Alberto M. Pereira:** Writing – review and editing. **Eric Fliers:** Writing – review and editing; supervision; conceptualization. **Dick F. Swaab:** Supervision; writing – review and editing; conceptualization. **Andries Kalsbeek:** Conceptualization; writing – review and editing; writing – original draft; supervision. **Chun‐Xia Yi:** Conceptualization; funding acquisition; writing – original draft; writing – review and editing; methodology; supervision; resources; project administration.

## FUNDING INFORMATION

This work was supported by an Amsterdam UMC PhD Scholarship (FCS, 2019) and the Dutch Diabetes Research Foundation (CXY, Diabetes Fonds, 2015.82.1826), The Netherlands.

## CONFLICT OF INTEREST STATEMENT

The authors have declared that no conflict of interest exists.

### PEER REVIEW

The peer review history for this article is available at https://www.webofscience.com/api/gateway/wos/peer-review/10.1111/jne.70015.

## Supporting information


**Figure S1.** Supporting Information.

**Figure S2.** Supporting Information.

**Figure S3.** Supporting Information.

**Figure S4.** Supporting Information.

**Figure S5.** Supporting Information.

**Data S1.** Supporting Information.

## Data Availability

The data that support the findings of this study are available on request from the corresponding author. The data are not publicly available due to privacy or ethical restrictions.
