# Supplementary figures and images for: Selective changes in vasopressin neurons and astrocytes in the suprachiasmatic nucleus of Prader–Willi syndrome subjects
Source: J Neuroendocrinol. 2025 Mar 8;37(5):e70015. doi: 10.1111/jne.70015 (PMC12045672; doi:10.1111/jne.70015)

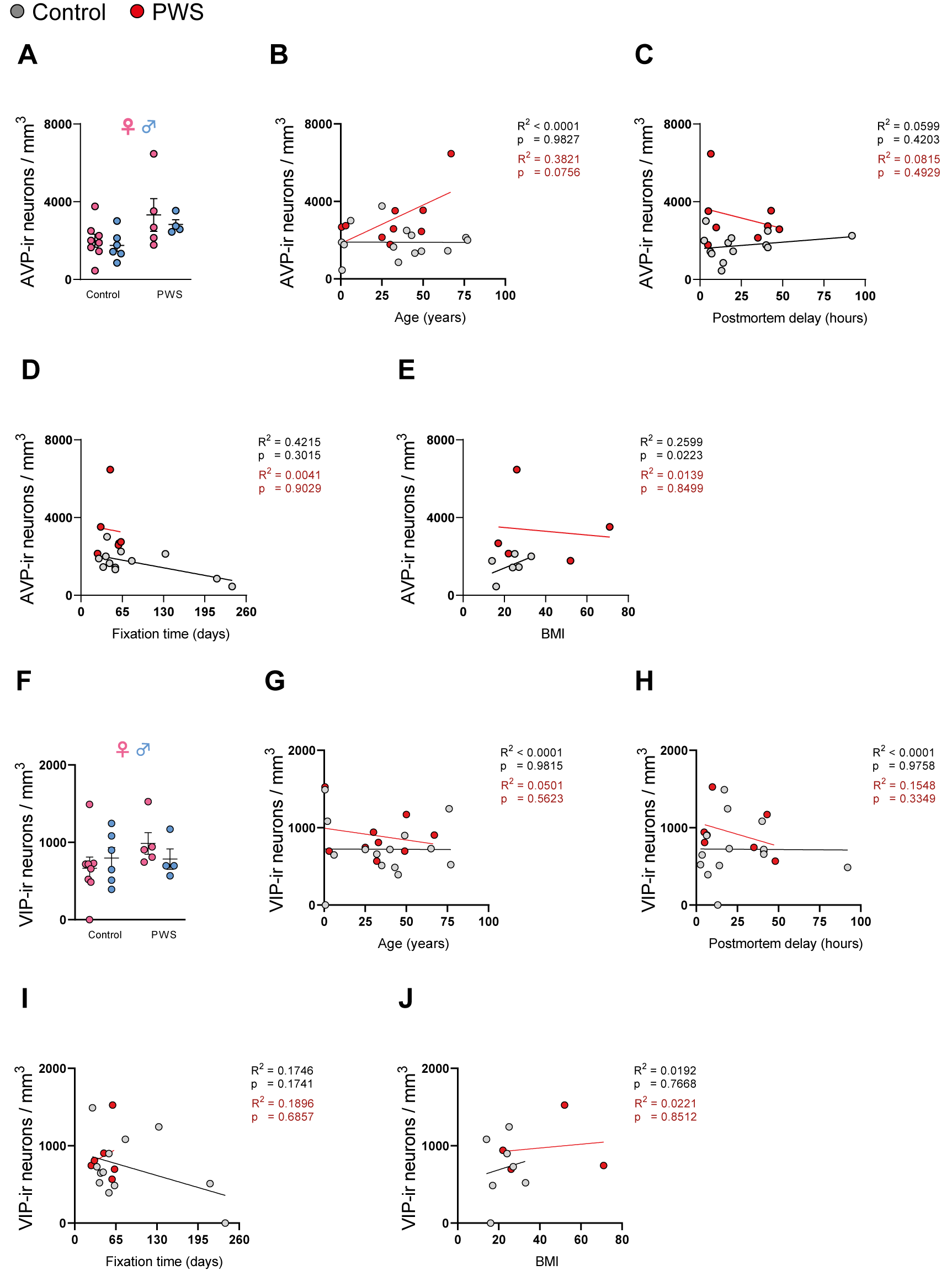

Supplement: Supplementary file 1 — Figure S1. Supporting Information. [file JNE-37-e70015-s001.tif]

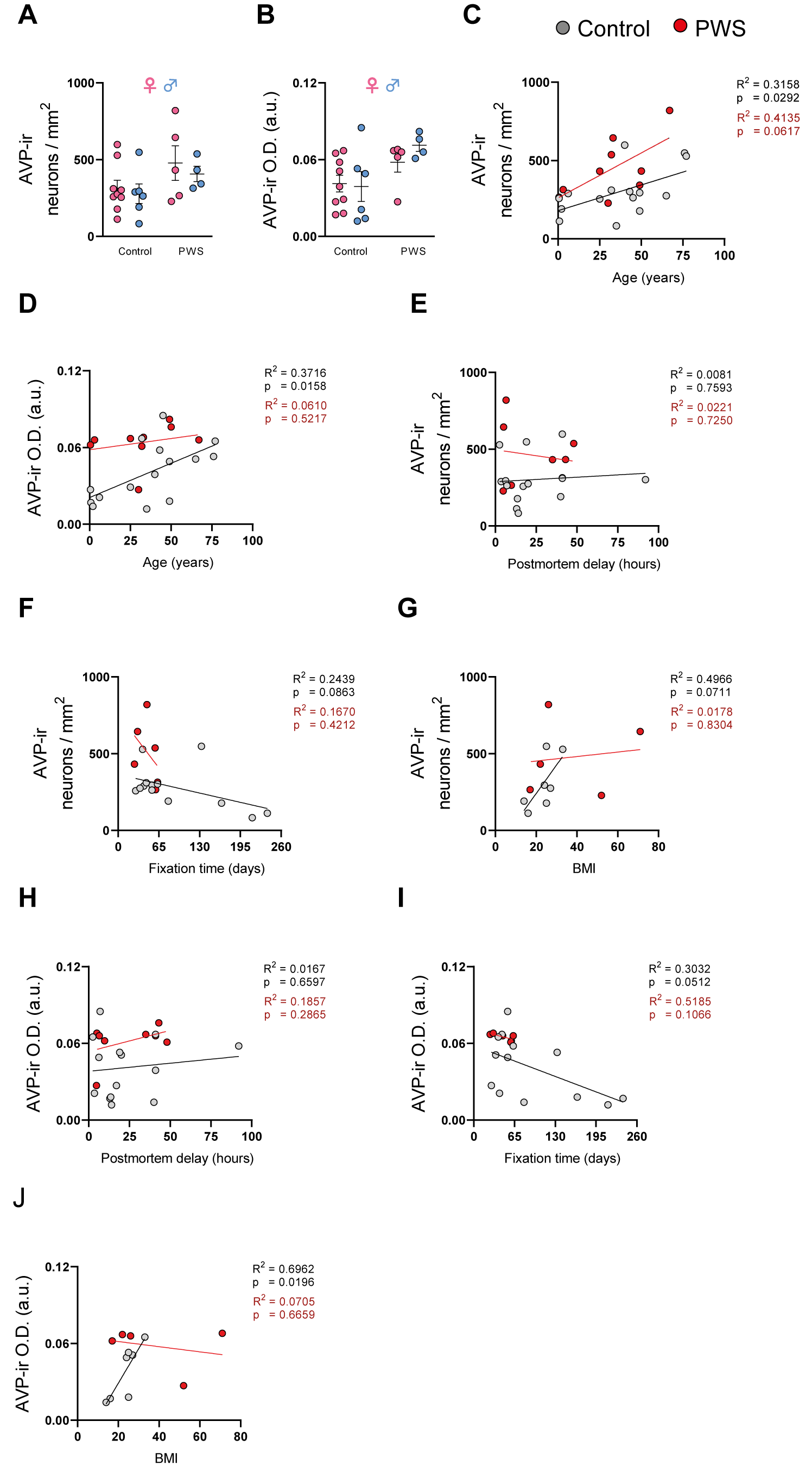

Supplement: Supplementary file 2 — Figure S2. Supporting Information. [file JNE-37-e70015-s002.tif]

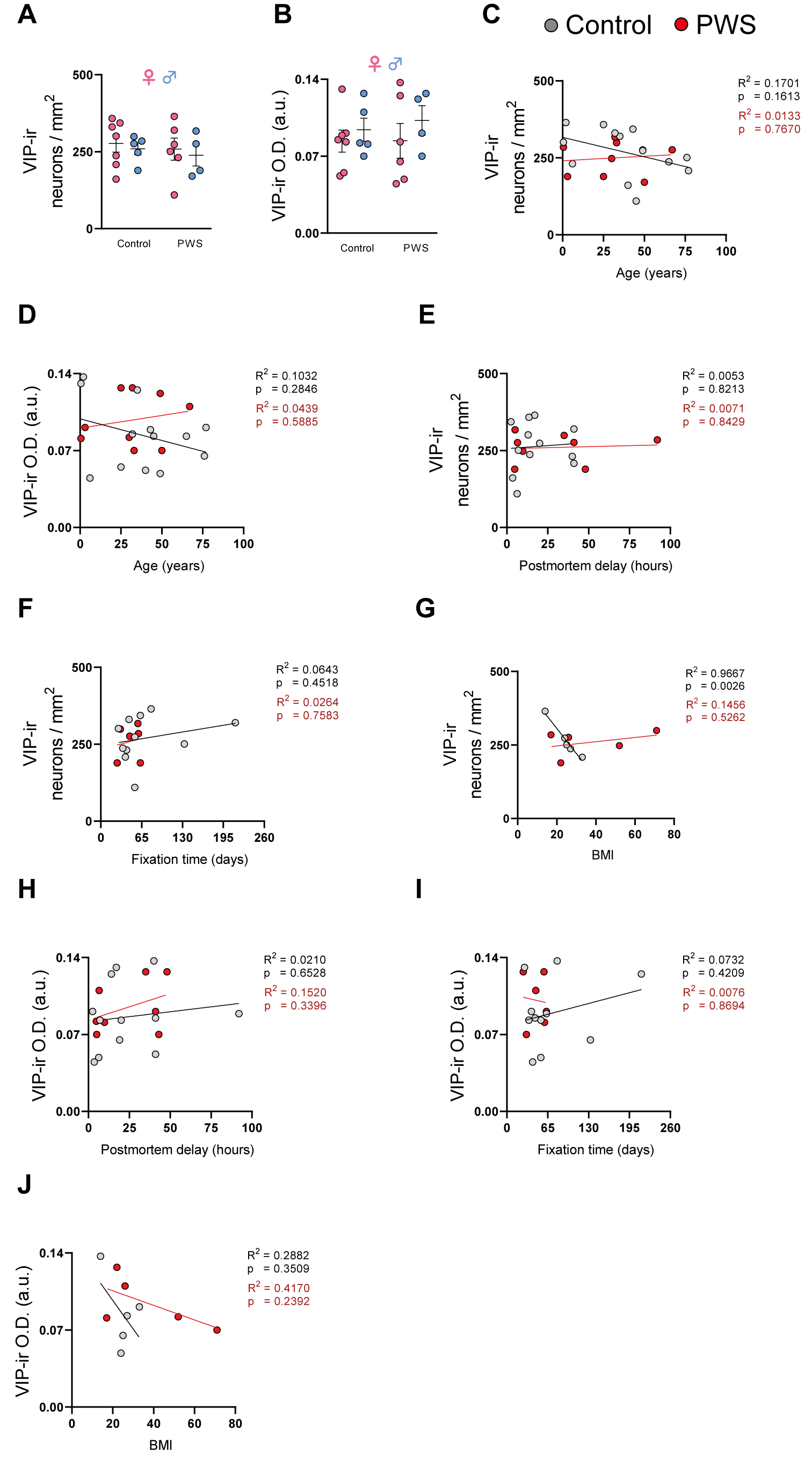

Supplement: Supplementary file 3 — Figure S3. Supporting Information. [file JNE-37-e70015-s004.tif]

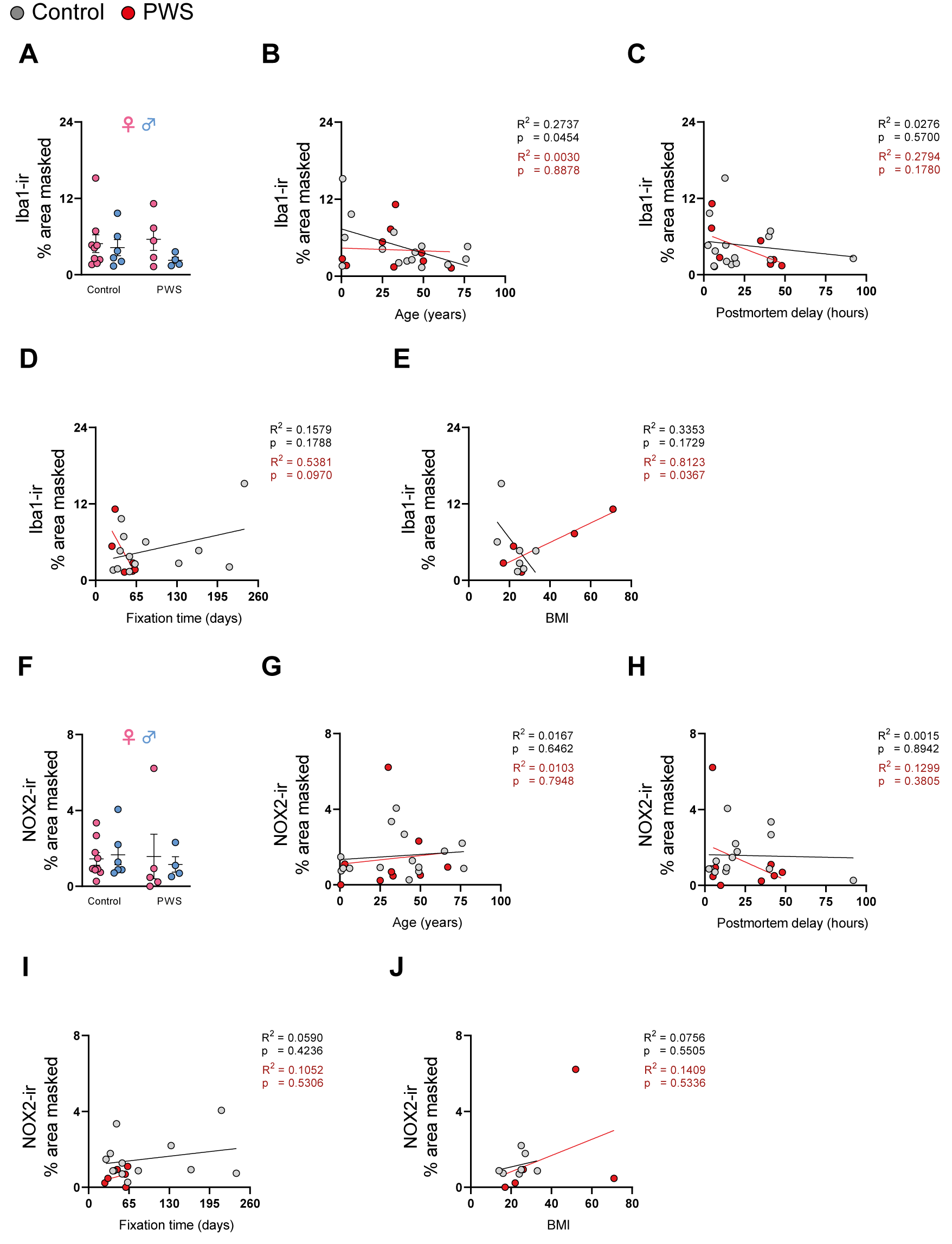

Supplement: Supplementary file 4 — Figure S4. Supporting Information. [file JNE-37-e70015-s003.tif]

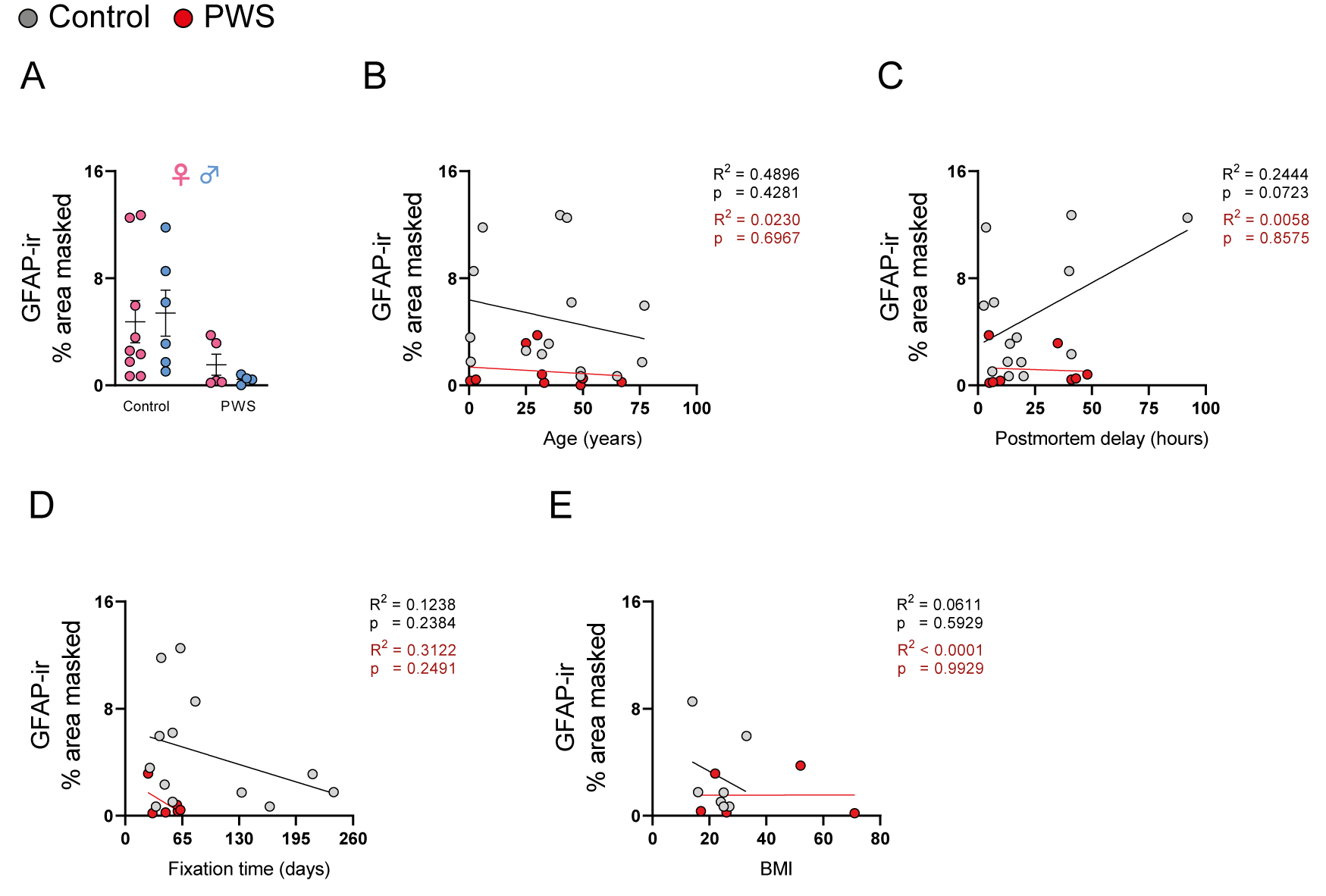

Supplement: Supplementary file 5 — Figure S5. Supporting Information. [file JNE-37-e70015-s006.tif]
