# Supplementary material for: Selective changes in vasopressin neurons and astrocytes in the suprachiasmatic nucleus of Prader–Willi syndrome subjects
Source: J Neuroendocrinol. 2025 Mar 8;37(5):e70015. doi: 10.1111/jne.70015 (PMC12045672; doi:10.1111/jne.70015)
Supplement: Supplementary file 6 — Data S1. Supporting Information. [file JNE-37-e70015-s005.docx]

**Selective changes in vasopressin neurons and astrocytes**

**in the suprachiasmatic nucleus of Prader-Willi Syndrome subjects**

Felipe Correa-da-Silva^1,2,3,4^, Jari B. Berhout^5^, Pim Schouten^1,2^, Margje Sinnema^6^, Constance T. R. M. Stumpel^6^, Leopold M.G. Curfs^7^, Charlotte Höybye^8^, Ahmed Mahfouz^9,10^, Onno C. Meijer^5^, Alberto M. Pereira^1,2^, Eric Fliers^1,2^, Dick F. Swaab^4^, Andries Kalsbeek^1,2,3,4^, Chun-Xia Yi^1,2,3,4 *^

1. Department of Endocrinology and Metabolism, Amsterdam University Medical Center, location AMC, University of Amsterdam, Amsterdam, The Netherlands.
2. Amsterdam Gastroenterology Endocrinology and Metabolism, Amsterdam, The Netherlands.
3. Department of Clinical Chemistry, Laboratory of Endocrinology, Amsterdam University Medical Center, location AMC, Amsterdam, The Netherlands.
4. Netherlands Institute for Neuroscience, Amsterdam, The Netherlands.
5. Dept. of Medicine Div. Endocrinology, Leiden University Medical Centre, Leiden, The Netherlands.
6. Department of Clinical Genetics, Maastricht University Medical Center, Maastricht, The Netherlands.
7. Governor Kremers Centre, Maastricht University Medical Centre, Maastricht, The Netherlands.
8. Department of Endocrinology and Department of Molecular Medicine and Surgery, Karolinska University Hospital and Karolinska Institute, Stockholm, Sweden.
9. Delft Bioinformatics Lab, Technical University Delft, Delft, The Netherlands.
10. Dept. of Human Genetics, Leiden University Medical Centre, Leiden, The Netherlands.

**Supplementary figures and legends**


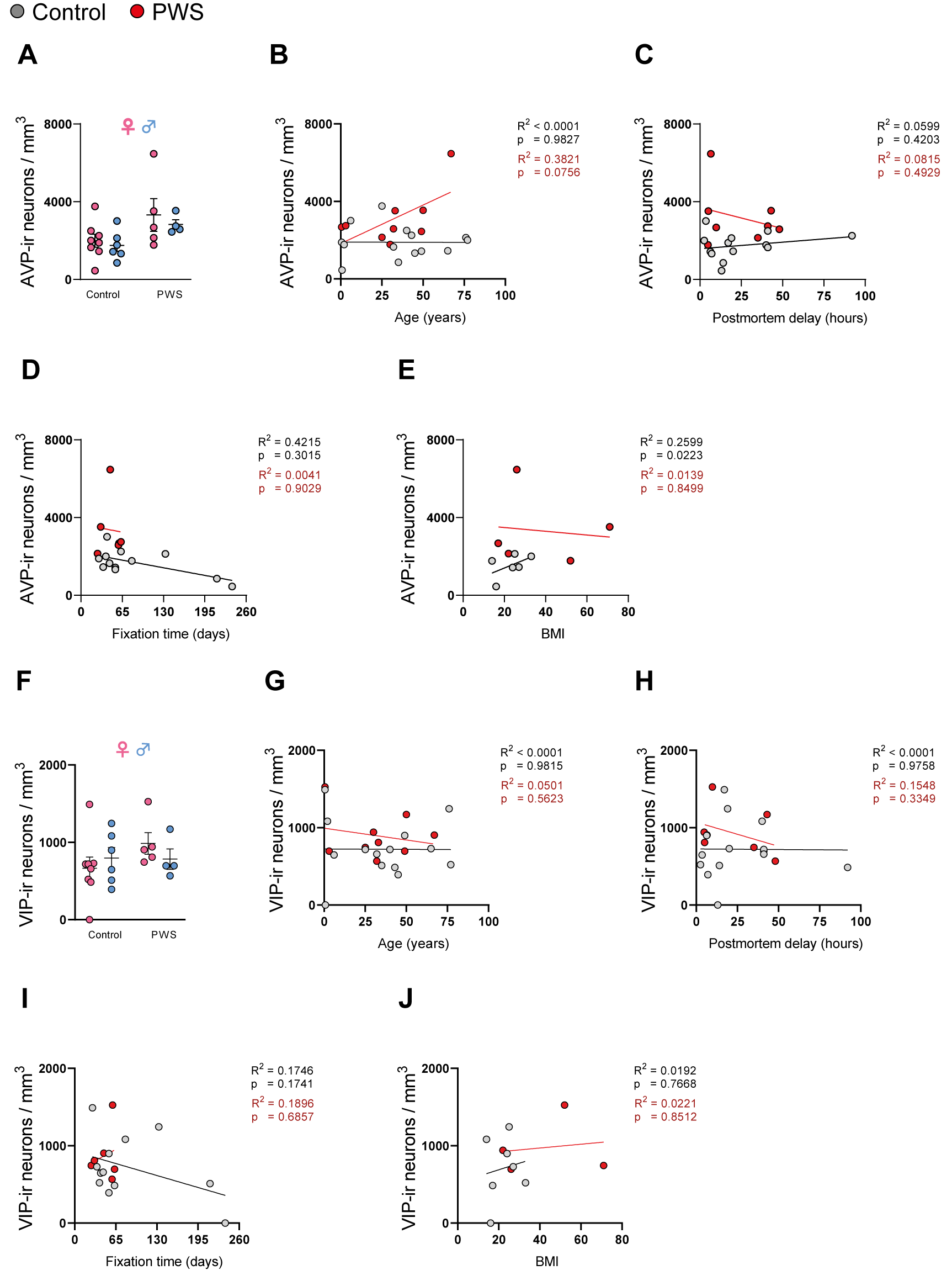


**Supplementary Figure 1.** Confounders analysis of AVP-ir and VIP-ir neurons in the SCN of control and PWS subjects. (A) Comparison of AVP-ir number between men and women. Plots of SCN AVP-ir number according to age (B), postmortem delay (C), fixation time (D) and BMI (E). (F) Comparison of VIP-ir number between man and woman. Plots of SCN VIP-ir number according to age (G), postmortem delay (H), fixation time (I) and BMI (J). Controls n=15; PWS n=9.


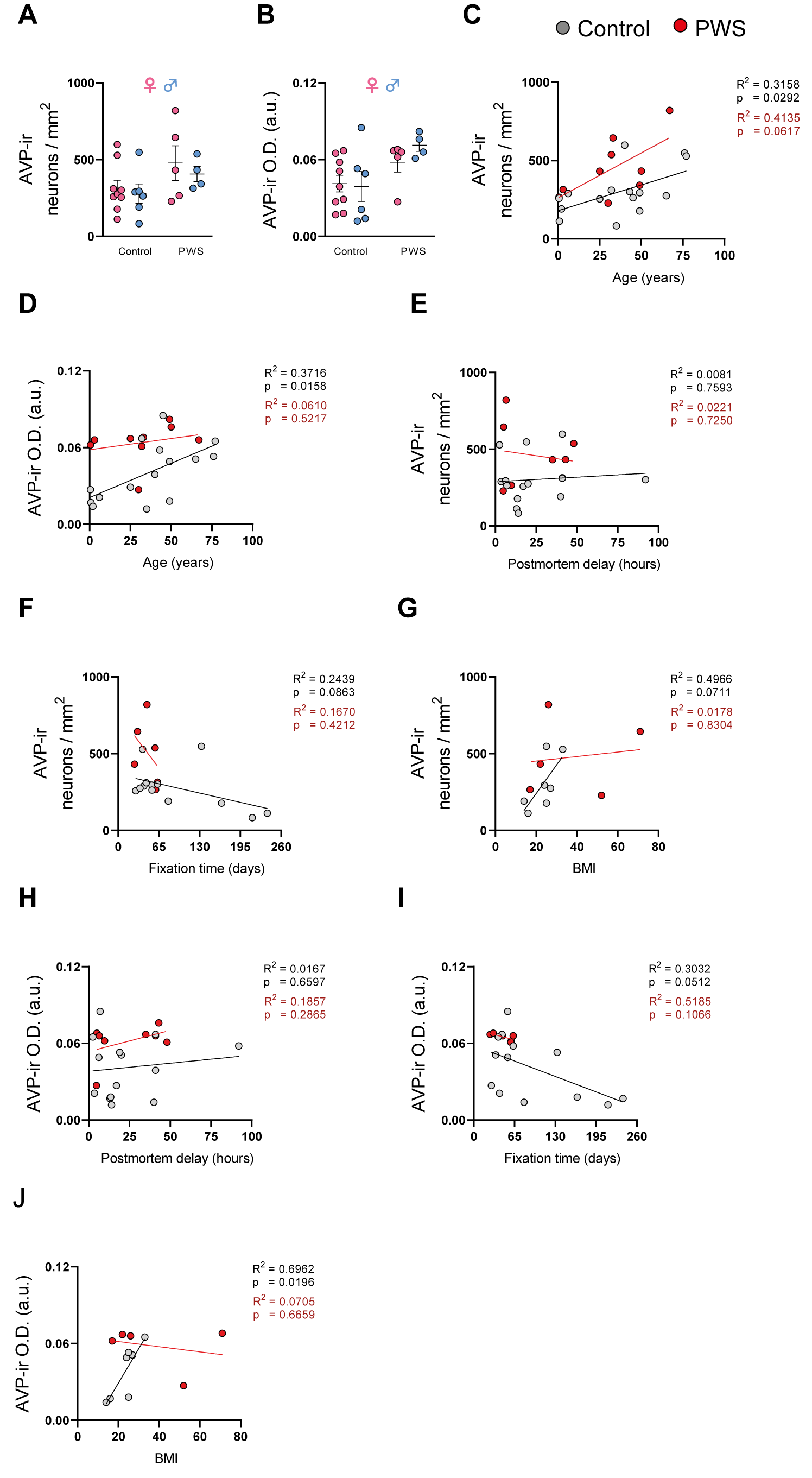


**Supplementary Figure 2.** Confounders analysis of AVP-ir neurons in the peak of the SCN of control and PWS subjects. (A-B) Comparison of AVP-ir soma number/mm^2^ and relative optical density between men and women. (C-D). Plots of SCN AVP-ir neuronal density and relative optical density according to age. Plots of AVP-ir neuronal density according to postmortem delay (E), fixation time (F) and BMI (G). Plots of AVP-ir relative optical density according to postmortem delay (H), fixation time (I) and BMI (J). Controls n=15; PWS n=9.


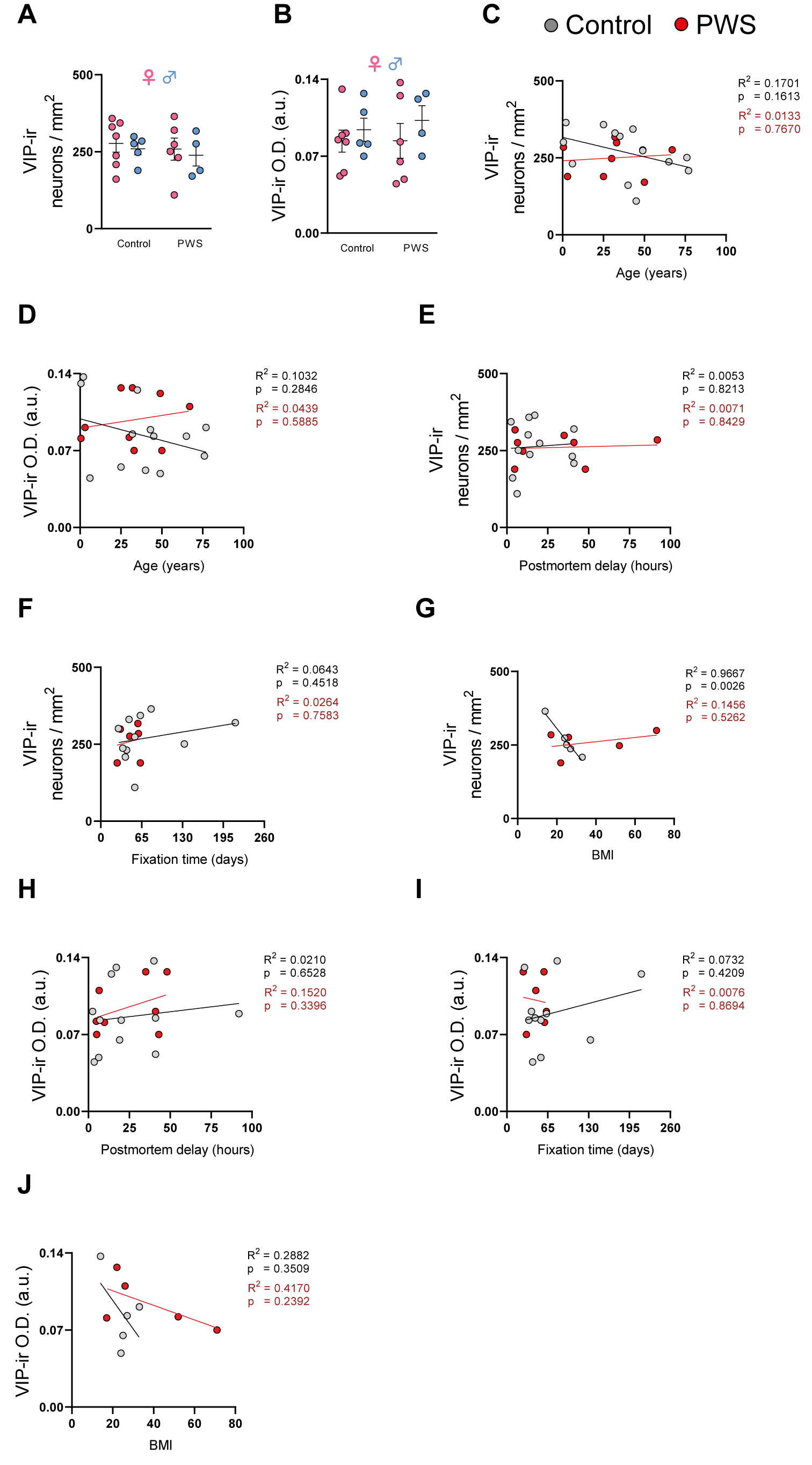


**Supplementary Figure 3.** Confounders analysis of VIP-ir neurons in the peak of SCN of control and PWS subjects. (A-B) Comparison of VIP-ir soma number/mm^2^ and relative optical density in relation between men and women. (C-D). Plots of SCN VIP-ir neuronal density and relative optical density according to age. Plots of VIP-ir neuronal density according to postmortem delay (E), fixation time (F) and BMI (G). Plots of VIP-ir relative optical density according to postmortem delay (H), fixation time (I) and BMI (J). Controls n=15; PWS n=9.


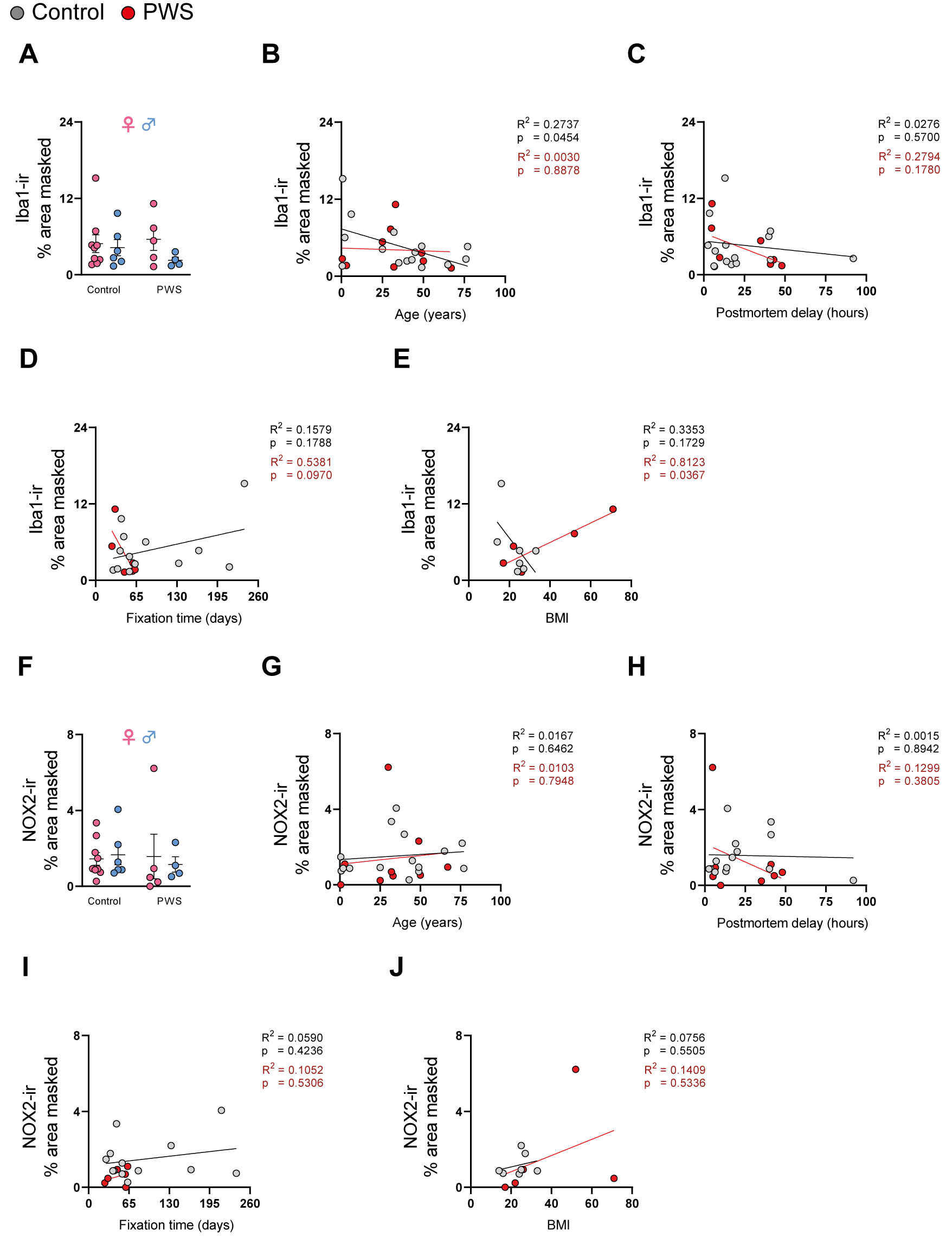


**Supplementary Figure 4.** Confounders analysis of microglia in the SCN of control and PWS subjects. (A) Comparison Iba1-ir relative area masked between men and women. Plots of SCN Iba1-ir relative area masked according to age (B), postmortem delay (C), fixation time (D) and BMI (E). (F) Comparison NOX2-ir relative area masked between men and women. Plots of SCN Iba1-ir relative area masked according to age (G), postmortem delay (H), fixation time (I) and BMI (J). Controls n=15; PWS n=9.


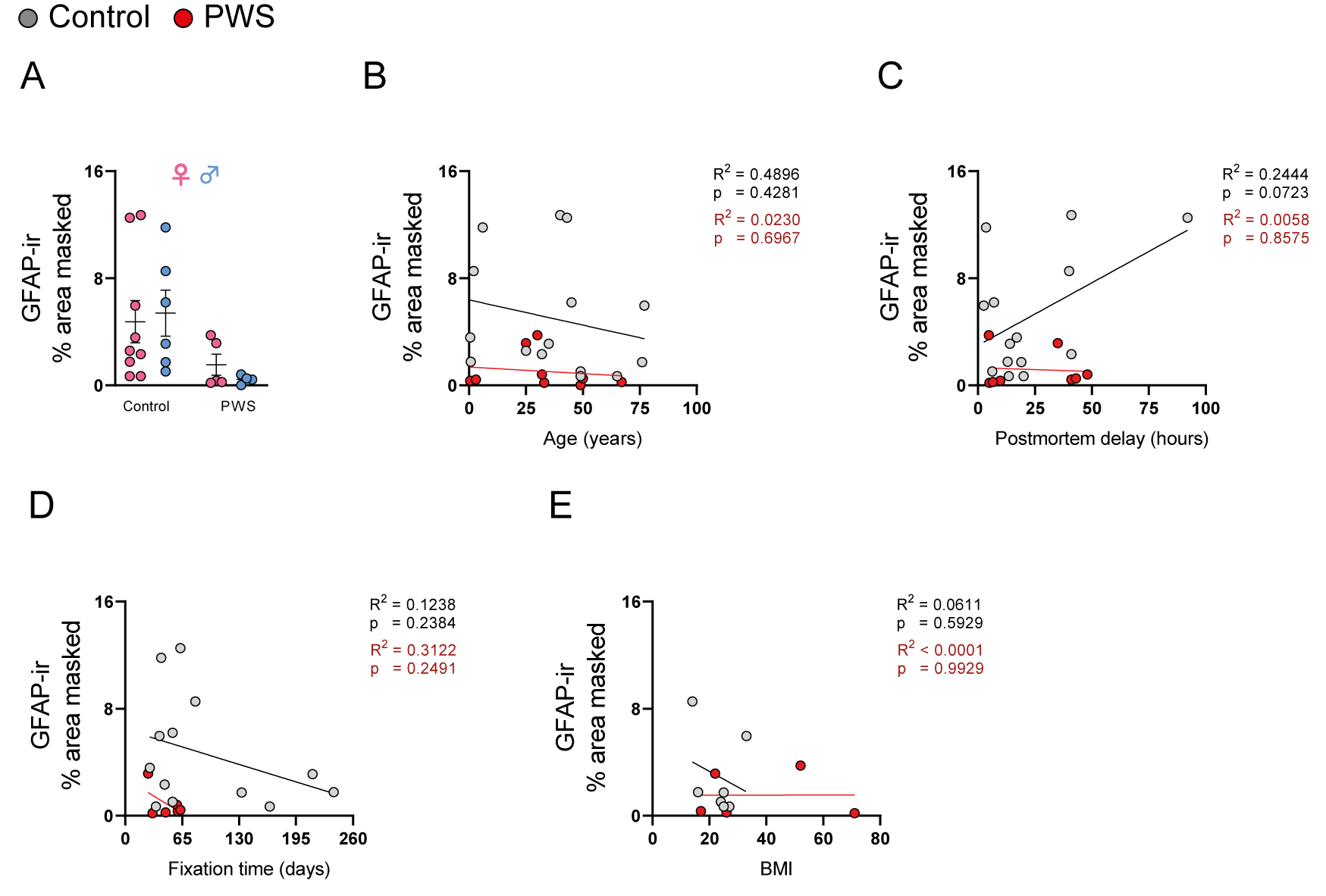


**Supplementary Figure 5.** Confounders analysis of astroglia in the SCN of control and PWS subjects. (A) Comparison of GFAP-ir relative area masked between men and women. Plots of SCN GFAP-ir relative area masked according to age (B), postmortem delay (C), fixation time (D) and BMI (E). Controls n=15; PWS n=9.
